# Vocal fold composition and early glottic carcinoma infiltration

**DOI:** 10.1186/1477-7819-10-178

**Published:** 2012-08-30

**Authors:** Qin Fang, Yang Wang, Xiaoyan Zhao, Luhong Cao, Na Sun, Xuejun Tan, Lide Wu, Guangbin Sun

**Affiliations:** 1 Department of Otolaryngology, Gongli Hospital, 219 Miaopu Road, Pudong New Area, Shanghai, 200135, China; 2 Department of Pathology, Changhai Hospital, Shanghai, 200433, China; 3 Department of Otolaryngology, Ningxia Medical University, Ningxia, 750004, China; 4 Department of Otolaryngology, Wanzhou Shanghai hospital, Chongqing, 404000, China

**Keywords:** Infiltration, Laryngeal carcinomas, Pathology, Surgery, Vocal fold

## Abstract

**Background:**

Current imaging techniques provide only limited information pertaining to the extent of infiltration of laryngeal carcinomas into vocal fold tissue layers. Therefore, it is needed to seek the contribute to the body of knowledge surrounding examination and characterization in laryngeal carcinoma infiltration.

**Methods:**

Excised larynges were collected from 30 male laryngectomy patients with an average age of 43.5 years (ranging 36 to 55 years) and history of smoking (≥10 years) exhibiting T1, T2, or subglottal (normal vocal fold) carcinomas. Vocal folds were preserved via freezing or immersion in paraffin. The depth of the mucosa, submucosa, and muscular layers in both normal vocal folds and tumor tissues of afflicted vocal folds was measured.

**Results:**

The average depths of the mucosa, submucosa, and muscular layers in normal vocal folds were 0.15 ± 0.06 mm, 2.30 ± 0.59 mm, and 2.87 ± 0.88 mm, respectively. Infiltration measurements of T1 tumors showed a depth of 1.62 ± 0.51 mm and 1.32 ± 0.49 mm in frozen sections and paraffin-embedded samples, respectively. Similarly, T2 tumors showed a depth of 2.87 ± 0.68 mm and 2.58 ± 0.67 mm in frozen sections and paraffin-embedded samples, respectively. T1 and T2 tumors occupied 24.8 ± 10 and 48.5 ± 15 percent of the normal vocal fold depth, respectively.

**Conclusion:**

This data provides a baseline for estimating infiltration of laryngeal carcinomas in vocal fold tissue layers, of particular interest to surgeons. This information may be used to assess typical depths of infiltration, thus allowing for more appropriate selection of surgical procedures based on individual patient assessment.

## Background

More effective treatment options are now available for patients suffering from early glottic cancer. Many of these treatments cost less and cause fewer side effects than previous therapeutic regimes. One such treatment option is laser excision, a procedure that preserves vocal quality without the requirement of a lengthy hospital stay [1-3]. Laser excision is inexpensive and has been reported to result in lower recurrence rates than radiotherapy treatment [4-7]. Additionally, numerous previous studies have validated laser surgery as a viable option for the treatment of select Tis, T1, and T2 cancers [8]. In one notable retrospective study detailing 15 years of data, Motta and colleagues [9] reported successful treatment of T1, T2, and even select T3 cancers using laser surgery. While laser excision surgery has been demonstrated to be effective, the parameters for examination and extent of tissue removal remain very subjective, which may limit the widespread implementation and success of this treatment method.

The extent of tissue removal is one of the most important indications of functional outcome following laser excision surgery or laser microsurgery treatments. Careful histological examination can be applied to confirm disease-free margins and limit the resection area, allowing for the maximization of preserved laryngeal tissue and better vocal preservation [9,10]. One study reported that a 2 mm margin was sufficient to generate a local cure rate of 95% in T1 carcinomas, indicating the feasibility of narrow-margin surgical techniques [10]. As laser surgeries increase in popularity, reliable documentation of the average extent of tumor invasion in different early glottic cancer classes and affected tissue layers is necessary to aid surgeons in procedure selection and design [11-13]. In order to remove cancerous tissues more efficiently, and with minimal damage to healthy neighboring tissues, further information must be documented and made readily available to clinicians interested in vocal fold composition and cancerous cell infiltration [14,15].

In this report, the composition of normal vocal folds and infiltration depths of tumor tissues within the vocal fold are measured and recorded. As tumor tissues were preserved via embedding in paraffin or freezing (methods that may induce tissue contraction and expansion [16]) actual depths were determined to be between these two measurements using light microscopic techniques. These findings will contribute to the development of an improved understanding and potentially publishable clinical standard that will be useful in determining the required resection area necessary for treating glottic carcinomas and related cancers of the vocal folds.

## Methods

### Patients and sample selection

Vocal cords and early glottic carcinoma tissues were obtained from 30 male laryngectomy patients with documented smoking habits for more than 10 years. Patients had an average age of 43.5 years, ranging from 36 to 55 years. Of the 30 patients enlisted in the study, 15 were treated for T1N0M0 glottic carcinoma and had previously undergone cordectomy procedures. Additionally, five patients had undergone partial laryngectomy for the treatment of T2N0M0 glottic carcinoma. The remaining 10 patients underwent total laryngectomy for the treatment of supraglottic carcinoma (five cases of T2N0M0 and three cases of T3N1M0) or subglottic carcinoma (two cases of T3N1M0) that evidenced no invasion of the glottic area or at least one remaining normal side of the vocal fold. These patients served as the source of normal vocal fold samples. All patients underwent treatment at the Department of Otolaryngology of Changhai Hospital. Samples were obtained in accordance with all applicable ethical and legal requirements, and the protocol was approved by the Ethics Committee of Changhai Hospital.

Notably, patients were excluded who had received radiotherapy or chemotherapy prior to surgery. The course of treatment for all patients included in the current study reflects the standard therapeutic approach applied by the Department of Otolaryngology at Changhai Hospital.

### Specimen collection

All samples were directly collected following surgical excision, and samples were managed by the same pathologist throughout the study. Other than the previously detected carcinoma, no other pathological abnormalities were reported. Isolated vocal folds were evenly segmented into three equal parts in the lengthwise direction (anterior, middle, and posterior), and each segment was further divided into two equal sections. As a result, six individual segments were obtained for analysis from each vocal fold. These vocal fold tissue segments were further sliced into 4 μm thick sections and treated with H&E staining. Of the six anterior, middle, and posterior sections generated from each sample, half (three sections) were selected for preservation in paraffin, and the remaining half (three sections) were frozen. Normal sections were made by isolating the middle segment of vocal folds from patients with supraglottic or subglottic carcinoma. All normal sections were sliced to 4 μm thickness, stained with H&E, and preserved in paraffin.

### Paraffin-embedded sample preparation

After segmentation, tissues to be preserved in paraffin were placed in 10% formalin for at least 48 hours. Samples were then dehydrated by immersion in graded ethanol solutions of progressively higher ethanol concentrations (50, 70, 85, 95 and 99% ethanol solution, respectively; 1 hour treatment at each concentration). Hyalinization was completed in dimethylbenzene. Resultant samples were placed into dissolved paraffin, heated to 60 to 62°C, inserted into slides using a Leica RM2135 paraffin microtome (Leica, Wetzlar, Germany), and dried in an oven at 45°C under normal air conditions. Prior to observation, sample tissues were deparaffinized in dimethylbenzene, rehydrated by immersion in graded ethanol solutions (high to low concentrations of 99, 95, 85, 70 and 50%. respectively; 1 hour treatment at each concentration), and finally immersed in distilled water. Next, samples were stained by brief immersion in a hematoxylin solution. Specimens were then moved to an acid and ammonia solution for several seconds. Samples were washed with running distilled water for 1 hour followed by brief immersion in distilled water. Samples were then transferred to 70% and 90% ethanol solutions for 10 minutes for dehydration. Eosin staining was conducted in an alcohol-eosin solution over the course of approximately 2 to 3 minutes. Final preparations for observation were completed by further dehydration in pure alcohol and hyalinization in dimethylbenzene. Lastly, a drop of gum was applied, and a glass coverslip was placed on each section for observation.

### Frozen sample preparation

Freezing was conducted using a Shandon Frozen Microtome (Shandon Scientific, Cheshire, England). Samples were placed in an optimal cutting temperature compound during freezing. Once frozen, specimens were further sliced into 4 μm thick sections. Prior to observation, tissues were allowed to thaw to room temperature and each section was placed on a slide. These sections were fixed with methanol for 10 to 20 seconds, washed, placed in hematoxylin for 1 minute, washed a second time, placed in hydrochloric acid and ethanol for 1 second, washed a third time, and briefly immersed in warm water. Thereafter, sections were placed in eosin for 2 to 5 seconds followed by quick dehydration in ethanol (low to high concentrations) and hyalinization with dimethylbenzene. Lastly, a drop of gum was applied, and a glass coverslip was placed on each section for observation.

### Measurements

**Figure 1:**
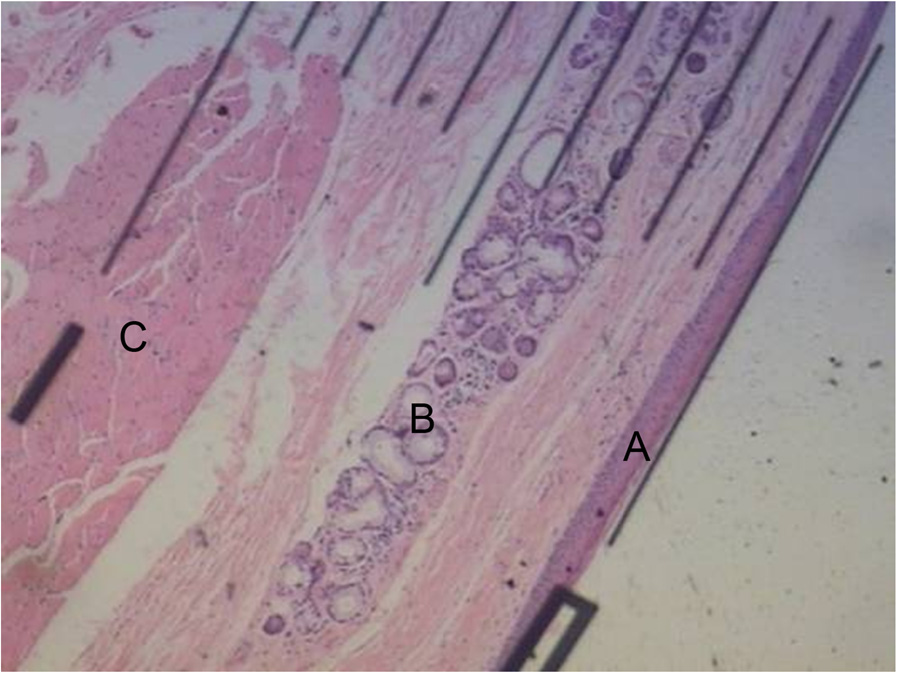
**Microscopic image of a normal vocal fold preserved by paraffinization and stained with H&E.** Marked regions indicate the (**A**) mucosa layer, (**B**) submucosa layer, and (**C**) muscular layer.

Specimens were examined using an Olympus BX-50 light microscope (Olympus, Tokyo, Japan) at a magnification of 40×. All measurements were made by a single, experienced examiner using a light micrometer in order to ensure operator consistency. Mucosa, submucosa, and muscular layer depths were recorded for 10 normal vocal fold samples. The extent of invasion by glottic carcinoma tissues was also measured for each section containing T1 or T2 carcinomas. No samples evidenced obscured or unclear boundaries between cancerous and normal tissue layers upon observation, resulting in a limited error range in these data (Figure 1). Individual measurements were recorded for frozen and paraffin-embedded samples.

### Statistical analysis

Means and standard deviations (mean ± SD) were calculated for mucosa, submucosa, and muscular layer depth in normal vocal folds. The total depth of the vocal fold was calculated by summing depths of the mucosa, submucosa, and muscular layers. Mean ± SD were determined for normal vocal fold depth.

Tumor invasion depths were calculated separately for paraffin-embedded and frozen sections. The depth of the invasive tumor was recorded for each subject and preservation method as the maximum depth obtained in all examined sections. Means ± SD were calculated for T1 and T2 carcinomas using both preservation methods. The percent of vocal fold invasion for T1 and T2 carcinomas was calculated by dividing the mean tumor depth (paraffin-embedded) by the calculated normal vocal fold depth.

## Results

### Normal vocal fold analysis

The average vocal fold depth of the mucosa, submucosa, and muscular layers was 5.32 ± 0.80 mm (Figure 1). The mucosa layer was the thinnest, spanning only 0.15 ± 0.06 mm of the total fold depth with a 95% confidence interval of 0.046. The submucosa and muscular layers comprised similar portions of the vocal fold, spanning 2.30 ± 0.59 mm and 2.87 ± 0.88 mm with 95% confidence intervals of 0.42 and 0.63, respectively (Table 1). The calculated average depth to the muscular layer was assumed to be equal to the sum of the depths of the mucosa and submucosa layers, resulting in a depth of 2.45 ± 0.61 mm (95% confidence interval 0.57).

**Table 1:** Width of normal vocal fold components measured using paraffin-embedding preservation

	Mucous layer width*	Submucosa layer width*	Muscular layer width*	Total width of vocal fold*
Mean ± SD	0.15 ± 0.06	2.30 ± 0.59	2.87 ± 0.88	5.32 ± 0.80
95% CI	0.046	0.42	0.63	0.57

*Values are shown in units of millimeters (mm). CI, confidence interval; SD, standard deviation.

### Carcinoma infiltration

T2 tumors were observed to occupy a larger portion of the vocal fold than T1 tumors in both the frozen and paraffin-embedded sections. On average, frozen samples exhibited larger cancer depth measurements than paraffin-embedded samples. The average depth of T1 tumor tissues in the vocal fold section was 1.62 ± 0.51 mm in frozen samples and 1.32 ± 0.49 mm in paraffin-embedded samples (95% confidence intervals 0.28 and 0.27, respectively; Table 2), indicating lesions within the epithelium and lamina propria. T2 tumor tissues occupied a depth of 2.87 ± 0.68 mm in frozen samples and 2.58 ± 0.67 mm in paraffin-embedded samples (95% confidence intervals 0.84 and 0.83, respectively; Table 2), indicating the presence of lesion extension through the submucosal layer to the muscular layer. On average, T1 tumors occupied 24.8 ± 10% of the total vocal fold, and T2 tumors infiltrated 48.5 ± 15% of the normal vocal fold.

**Table 2:** Measured width of tumor tissues in glottic carcinoma samples by region

	**Anterior**	**Middle**	**Posterior**	**Maximum**
**Frozen samples**				
T1 carcinoma				
Mean ± SD*	1.18 ± 0.52	1.51 ± 0.53	1.43 ± 0.34	1.62 ± 0.51
95% CI	0.29	0.30	0.19	0.28
T2 Carcinoma				
Mean ± SD*	2.87 ± 0.68	1.95 ± 0.64	1.86 ± 0.17	2.87 ± 0.68
95% CI	0.84	0.80	0.22	0.84
**Paraffin-embedded samples**				
T1 Carcinoma				
Mean ± SD*	0.90 ± 0.52	1.2 ± 0.52	1.12 ± 0.33	1.32 ± 0.49
95% CI	0.29	0.29	0.18	0.27
T2 Carcinoma				
Mean ± SD*	2.58 ± 0.67	1.90 ± 0.34	1.58 ± 0.15	2.58 ± 0.67
95% CI	0.83	0.43	0.18	0.83

*Values are shown in units of millimeters (mm). CI, confidence interval; SD, standard deviation.

## Discussion

By accurately determining the extent of infiltration of tumor tissues of the vocal folds, surgeons can assess disease-free margins and preserve increased areas of the healthy laryngeal tissue. The current examination of vocal fold tumor penetration yielded depths comparable to those observed in previous research. While laser surgery has been increasingly used to treat both benign lesions and malignant tumors of the larynx, the method has been shown to be particularly effective for treating early-stage glottic carcinomas [4,5,7-10]. Additional documentation of vocal fold composition in the area of the surgical margins may allow surgeons to more accurately predict both the feasibility and outcome of such surgical procedures. Despite the value of this information, few previous studies have provided direct measurements of tumor infiltration, making these data critical to the development of standards for use in clinical practice. Such standards will be of particular importance as revision with laser surgery becomes more widely implemented as a tool for treating glottic cancer.

In his *Atlas on the Surgical Anatomy of Laryngeal Cancer*, Kirchner provided the initial groundwork for conservative laryngeal surgical procedures through his detailed descriptions and illustrations of laryngeal cancer cases over a 25-year period [17]. This work was novel in that it focused on the growth patterns and spread of cancerous tissues within the larynx, parameters that have since come to be recognized as critical elements in the successful conservation of healthy tissues during surgery. Other researchers, such as Steiner and Ambrosch [18], have provided more modern surgical adaptations of these methods through endoscopy with the use of carbon dioxide lasers. This method allows water to absorb particular frequencies of light, minimizing extraneous damage to neighboring structures and normal tissues. A number of variations of such laser surgeries have been recently reported, suggesting that laser surgery may become an important tool in the conservative treatment of laryngeal cancers.

The current examination of normal vocal folds revealed a calculated total depth of 5.32 ± 0.80 mm, a slightly elevated but comparable result to that previously described in similar patient groups. One such report established a normal vocal fold depth range from 2.2 to 6.0 mm in adult males [19]. Another study demonstrated variation in vocal fold depth along the anterior-posterior axis of the vocal fold, revealing similar results of 5.69 mm and 4.83 mm for the right and left vocal folds, respectively [20]. Notably, the mucosal layer comprises only a minor portion of the total vocal fold depth (0.15 ± 0.06 mm) with the remaining depth being divided approximately equally between the submucosal and muscular layers, though the muscular layer consistently possesses slightly greater depths than the submucosal layer (2.30 ± 0.59 mm and 2.87 ± 0.88 mm, respectively).

In the current study, T1 tumors showed limited infiltration, with a depth of only 1.32 ± 0.49 mm in paraffin-embedded tissues and 1.62 ± 0.51 mm in frozen tissues. These depths were well short of achieving muscular layer breach, which rarely occurs at depths shallower than 2.45 ± 0.59 mm (sum of mucosa and submucosa layer depths). Thus, T1-staged tumors are generally limited to the vocal fold and will not impair vocal fold abduction, indicating that T1 tumors are constrained to the submucosa layer [21]. Conversely, some studies have indicated contradictory findings, including a study by Kocatürk and colleagues [21] that showed muscle invasion in 5 of 16 T1 subjects (tumor depth of 2 to 3 mm). Manola and colleagues [10] also recorded a maximum average tumor depth of 2.18 mm for T1 glottic carcinomas, suggesting that tumors may have breached the muscular layer. Discrepancies in T1 infiltration depths may be due to contraction or expansion during preservation, making direct comparison between studies using different preservation methods difficult. When frozen, samples tend to expand, such that, even with rapid freezing, tissues are reported to enlarge by 6%. Formalin fixation and dehydration causes even more drastic changes in tissue size, resulting in shrinking of up to 30% [21]. Thus, the actual measurements were assumed to exist between these two measurements in the present study. Alternatively, the current results may be indicative of less advanced cancer stage, potentially reflecting a conservative staging approach, or variation due to relatively small cohort sizes.

The extent of T2 tumor infiltration remains largely undocumented. Current findings, however, demonstrated that T2 tumors were more advanced, as indicated by elevated invasiveness. The infiltration depth of T2 tumors was 2.87 ± 0.68 mm in frozen samples and 2.58 ± 0.67 mm in paraffin-embedded samples, suggesting that the average T2 tumor breeched the muscular layer. Thus, T2 tumors were determined to be slightly more invasive than T1 tumors. Further investigation of T2 and T3 tumors may also provide improved guidelines for determining optimal surgical approaches, including determination of which tumors are most appropriately resected using CO_2_ laser surgery.

Despite the importance of tumor characterization prior to surgery, surgeons generally are able to attain only limited information regarding the extent of tumor infiltration. Past studies have shown that tumor volume, muscular and cartilaginous invasiveness, nodal disease, infiltration into the supraglottic, glottis, and subglottic compartments, and soft tissue dissemination all contribute to treatment outcome [4,22]. In order to precisely determine tumor infiltration, Manola and colleagues [10] employed a combination of measures to successfully resect tumors with a 2 mm margin in 90.4% of cases. Unfortunately, most surgeons do not have access to such extensive and expensive pre-surgical testing equipment. While magnetic resonance imaging (MRI) has been shown to be superior to computerized tomography (CT) scans, overestimation of cartilage involvement is a common cause of negative outcomes related to swallowing, breathing, and pulsation artifacts. Similarly, CT and three-dimensional Fourier Transformation MRI were suggested to be the best systems for assessing tumor volume [23]. Despite recent advances, current imaging techniques fail to provide the complete picture of the carcinoma invasiveness necessary for conservative surgery.

Since imaging techniques provide only limited data pertaining to tumor infiltration, more precise measurements of tumor depth, such as those described in the current report, may provide valuable information to surgeons. The standard proposed by the current research suggests that T1 tumors generally reach a depth of 1.5 mm, allowing surgeons to rapidly excise tissues to this initial depth before slowly resecting remaining portions of the tumor. Additionally, pre-surgical estimation of necessary total vocal fold depth resection size may allow for improved predictions of post-operative vocal outcomes [7]. The present data represent only initial investigation, and further research will be necessary to generate more accurate measurements prior to clinical implementation. Future studies with larger cohort sizes should examine T1, T2, T3, and T4 tumor types before confirming clinical standards for widespread implementation.

Increased information about normal vocal folds and tumor infiltration depths could allow surgeons to not only predict surgical outcomes more accurately, but also to select ideal candidates for surgery. Muscular infiltration is one of the determinants of operative success and functional outcome. Furthermore, laser surgery is anatomically limited by the thyroid cartilage [4,22]. Based on tumor depth and vocal fold composition, surgeons may be able to more accurately anticipate the breach of these anatomical landmarks prior to surgery. In this study, the combined depth of the mucosa and submucosa layers was found to be 2.45 mm on average. Based on the lower limit of the confidence interval, tumors extending less than 1 mm into the vocal fold have a low probability of breaching the muscular layer, making these tumors ideal candidates for microsurgery. A reliable estimation of surrounding cartilage depth may, likewise, be used to predict oncological effectiveness, though additional research is required to validate this hypothesis. As such, functional outcomes are likely to be closely related to the depth of tumor penetration and size of resection necessary.

A remaining limitation of laser microsurgery is the precision of measurements that can be achieved during resection. Although the current data provides measurements at increments of 0.01 mm, current operating microscopy does not allow for this level of specificity. Rather, these measurements must be taken as a general estimation of the necessary excision size. As operating microscopes improve, however, such precise measurements may become increasingly useful. While current technology limits the utility of these data (providing measurements that are, at best, in the order of 0.1-0.5 mm), these data still provide improved suggestions for initial excisions that may improve the care afforded to individual patients.

## Conclusion

By defining the composition of the normal vocal fold and providing an estimation of the infiltration depths of T1 and T2 carcinomas, the current research provides additional information for surgeons undertaking resection of laryngeal tumor tissues. While current imaging techniques provide only limited information pertaining to tumor infiltration, the current investigation provides a standard of highly precise values for the characterization of tumor depth during early stage glottic carcinoma. The depth of different anatomical landmarks and tumor invasiveness can help surgeons to more accurately determine the excision size and avoid structural breaches. Additional research will, however, be required in order to more precisely define the extent of T1 and T2 tumors and the depth of vocal fold tissue layers by region. These data may directly increase the reliability of laser surgery treatment methods. Also, definition of variant T3 carcinoma infiltration may also be required to further expand the application of laser microsurgery in later-stage cancers.

## Abbreviations

CT: computerized tomography; H&E: hematoxylin and eosin; MRI: magnetic resonance image; SD: standard deviation.

## Competing interests

The authors declare that they have no competing interests.

## Authors’ contributions

Qin Fang collected the samples and drafted the manuscript, Yang Wang made the frozen and paraffin-embedded sections, Xiaoyan Zhao , Luhong Cao ,Xuejun Tan and LideWu collected the samples, Na Sun carried out the data analysis, Guangbin Sun designed the study, made the measurement and drafted the manuscript. All authors read and approved the final manuscript.

## Funding

The current work was supported by funding from The Fund of the Advanced Medical Department of the Pudong New Area, Shanghai (No. PWZxkq 2010–02) and the Shanghai Natural Science Fund (No. 10ZR1427200). The grantors had no direct involvement in the conception, execution, or reporting of this research.
